# Mutations of epigenetic genes and correlation with treatment response in peripheral T‐cell lymphoma

**DOI:** 10.1002/ctm2.1491

**Published:** 2024-01-18

**Authors:** Chong Wei, Wei Wang, Wanying Li, Yan Zhang, Danqing Zhao, Wei Zhang, Daobin Zhou

**Affiliations:** ^1^ Department of Hematology Peking Union Medical College Hospital Chinese Academy of Medical Sciences and Peking Union Medical College Beijing China; ^2^ Geneplus‐Beijing Beijing China

Dear Editor,

Peripheral T‐cell lymphomas (PTCLs) are heterogeneous and relatively rare diseases, which account for 20% to 30% of non‐Hodgkin's lymphoma in China.^1^, ^2^ Due to a lack of prospective studies and targeted therapeutic drugs, the treatment of PTCL is still disappointing. Long‐term survival of most types of PTCLs were around 30%–40%.^3^ Recent molecular studies revealed that PTCL with T‐follicular helper (TFH) origin including angioimmunoblastic T‐cell lymphoma (AITL) and nodal PTCL with TFH phenotype (nTFHL) share recurrent mutations in epigenetic regulating genes, including *TET2*, *DNMT3A* and *IDH2*.^4^, ^5^ Therapies targeting epigenetic changes, such as demethylation drugs and histone deacetylase (HDAC) inhibitors, are being investigated.^6^, ^7^ However, there is still a lack of evidence to determine the correlation between epigenetic mutations and the efficacy of epigenetic targeting drugs. In this prospective study, we aimed to evaluate the correlation between epigenetic mutations and treatment response and their prognostic value.

This was a prospective single‐center study. Key eligibility criteria included the following: (1) newly diagnosed PTCLs with pathological findings consistent with PTCL, not otherwise specified (PTCL‐NOS), AITL, and nTFHL based on the 4th edition of World Health Organization classification^2^; (2) enough formalin fixed paraffin embedded (FFPE) samples available for next‐generation sequencing (NGS); (3) enrolled in one of the two prospective trials: the first was the phase 1/2 trial of chidamide (also known as tucidinostat) combined with cyclophosphamide, vincristine, doxorubicin, etoposide and prednisone regimen (CHOEP), so‐called C‐CHOEP regimen in newly diagnosed PTCLs (NCT02987244)^8^; The second was the phase 3 trial of chidamide, azacitidine combined with cyclophosphamide, vincristine, doxorubicin and prednisone regimen (AC‐CHOP) versus CHOP regimen in newly diagnosed PTCLs (NCT05075460).^9^ Between January 2016 and April 2023, 38 patients were prospectively enrolled, including 15 patients in the phase 1/2 trial of C‐CHOEP regimen and 23 patients in the experiential arm of the phase 3 trial of AC‐CHOP regimen. A flowchart of patients from screening to analysis is shown in Figure S1. The base‐line clinical characteristics of the 38 patients are shown in Table 1.

**TABLE 1:** Clinical characteristics and treatments of the patients.

Characteristic	Number of patients (%)
Age, years	
Median (range)	58 (31−70)
>60	19 (50.0%)
Sex, male	24 (63.2%)
Ann Arbor stage III/IV	36 (94.7%)
B symptom present	30 (78.9%)
ECOG performance status > 1	14 (36.8%)
Elevated LDH level	28 (73.7%)
EBV‐DNA ≥ 500 copies/mL	16/35 (45.7%)
Bone marrow involvement	13 (34.2%)
No. of extranodal site > 1	14 (36.8%)
IPI score > 2	29 (76.3%)
PIT score > 1	24 (63.2%)
Histologic subtypes	
AITL	21 (55.3%)
nTFHL	5 (13.6%)
PTCL‐NOS	12 (31.6%)
Treatment regimens	
Chidamide+CHOEP	15 (39.4%)
Azacitidine+chidamide+CHOP	23 (60.5%)
Auto‐SCT consolidation	7 (18.4%)
Allo‐SCT consolidation	1 (2.6%)

Abbreviations: AITL, angioimmunoblastic T‐cell lymphoma; Allo‐SCT, allogeneic stem cell transplantation; Auto‐SCT, autologous stem cell transplantation; CHOEP, cyclophosphamide, doxorubicin, vincristine, prednisone and etoposide; CHOP, cyclophosphamide, doxorubicin, vincristine, and prednisone; EBV, Epstein‐Barr virus; ECOG, Eastern Cooperative Oncology Group; IPI, International Prognostic Index; LDH, lactate dehydrogenase; nTFHL, nodal peripheral T‐cell lymphoma with TFH phenotype (nTFHL); PTCL‐NOS, peripheral T‐cell lymphoma, not otherwise specified.

NGS of the 38 patients was performed on FFPE tissue samples using 1 of the 2 custom panels. The first custom panel included 413 lymphoma‐associated genes (Oncolym Panel, Geneplus‐Beijing, Beijing, China). The other custom panel included 84 genes that are frequently mutated in PTCLs (PTCL Panel, Yuanqi Bio, Shanghai, China). Gene lists of the two panels are provided in the supplementary materials (Tables S1 and S2). Both two panels included genes involved in DNA methylation (*TET2*, *IDH2 and DNMT3A*), histone methylation (*KMT2A*, *KMT2C*, *KMT2D*, *EZH2*, *BCOR* and *SETD2*), histone acetylation (*EP300* and *CREBBP*) and also other recurrent mutations in PTCLs (including *RHOA*, *PLCG*, *CD28*, *VAV1*, *FYN*, *STAT3* and *TP53*). Sequencing was performed on the HiSeq 3000 system. The average sequencing depth was 1153.6 ± 570.3. Buccal mucosa was obtained and sequenced to filter out germline mutations. Only mutations with variant allele frequencies of ≥1% were included in our analysis. Other statistical methods were as follows. Differences of continuous variable and categorical variable were estimated using the Mann–Whitney *U* test and the Fisher's exact test. Progression‐free survival (PFS) and overall survival (OS) were analyzed using the Kaplan‐Meier method. Survival rates differenced were compared using the Log‐rank test. Univariate analysis was performed using Cox regression for PFS and OS.

In total, 253 somatic mutations involving 109 genes were identified. The average number of mutations was 6.7 (range: 1–21) per sample. Mutational profile revealed from the 38 PTCL tumour samples were shown in Figure 1. Widespread mutations in epigenetic modifying genes were identified in this cohort. In line with previous studies, the most frequent epigenetic mutations were *TET2* mutations identified in 27/38 (71.1%) of the patients, *DNMT3A* mutations in 9/38 (23.6%) of the patients, *IDH2* mutations in 8/38 (21.0%) of the patients and *KMT2C* mutations in 6/38 (15.8%) of the patients. Among other recurrent mutations in PTCLs, *RHOA* mutations were identified in 20/38 (52.6%) of the patients and *PLCG1* identified in 5/38 (13.2%) of the patients. A total of 47 *TET2* mutations were identified in the 38 PTCL tumour samples (Figure 2A). All of the *IDH2* mutations occurred in the hotspot point (p.R172M/G/S/K) (Figure 2B). Most of the *RHOA* mutations occurred in the hotspot point (c.50G > T, p.G17V) except for two missense mutations (c.482C > T and c.358G > T) identified in one patient (ID32) (Figure 2C). AITL and nTFHL have higher frequency of *TET2*, *IDH2*, and *RHOA* mutations than in PCTL‐NOS (*TET2*: 80.8% vs. 50.0%, *p* = .052; *IDH2*: 30.8% vs. .0%, *p* = .039; *RHOA*:69.2% vs. 16.7%, *p* = .008). Mutations of *TET2*, *RHOA* and *IDH2* showed strong correlations. All but two *RHOA*‐mutated cases also harbored *TET2* mutations, and all of the *IDH2* mutations were identified exclusively in samples that also harbor *RHOA* mutations (Figure 2E).

**FIGURE 1:**
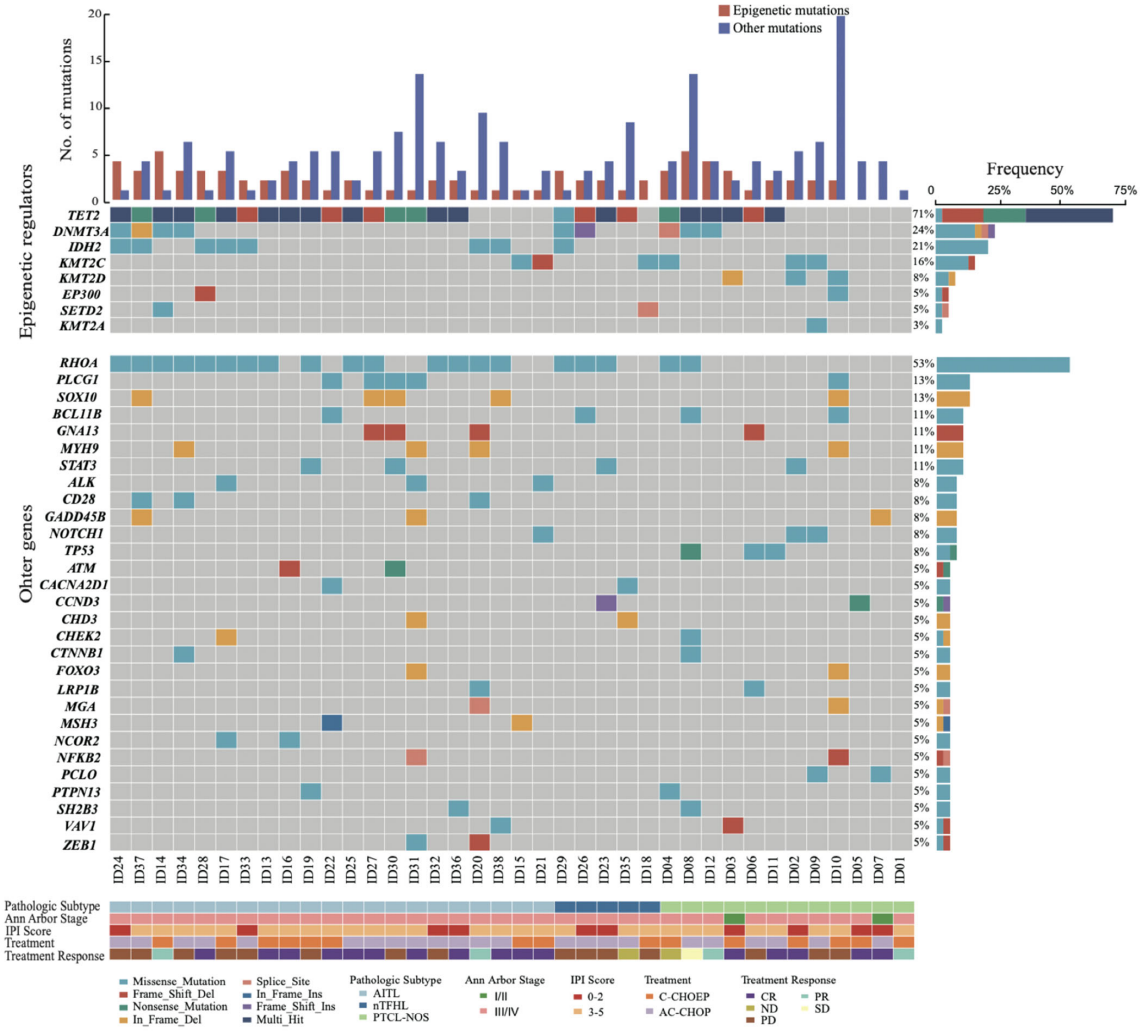
Mutational profile of the 38 patients with peripheral T‐cell lymphomas (PTCLs). The upper part represents mutations of epigenetic regulators including *TET2*, *DNMT3A*, *IDH2*, *KMT2A*, *KMT2C*, *KMT2D*, *SETD2* and *EP300*. The lower part represents other recurrent mutations restricted to genes mutated in more than 5% of the cases in this PTCL cohort. Each column represents one tumour sample, and each row represents one gene. The upper bars represent the number of epigenetic and other mutations in each patient. The right bars represent the frequencies of specific genes in the cohort. Pathologic subtype, Ann Arbor stage, IPI score, treatment and response are also shown. Cases are clustered according to pathological subtypes.

**FIGURE 2:**
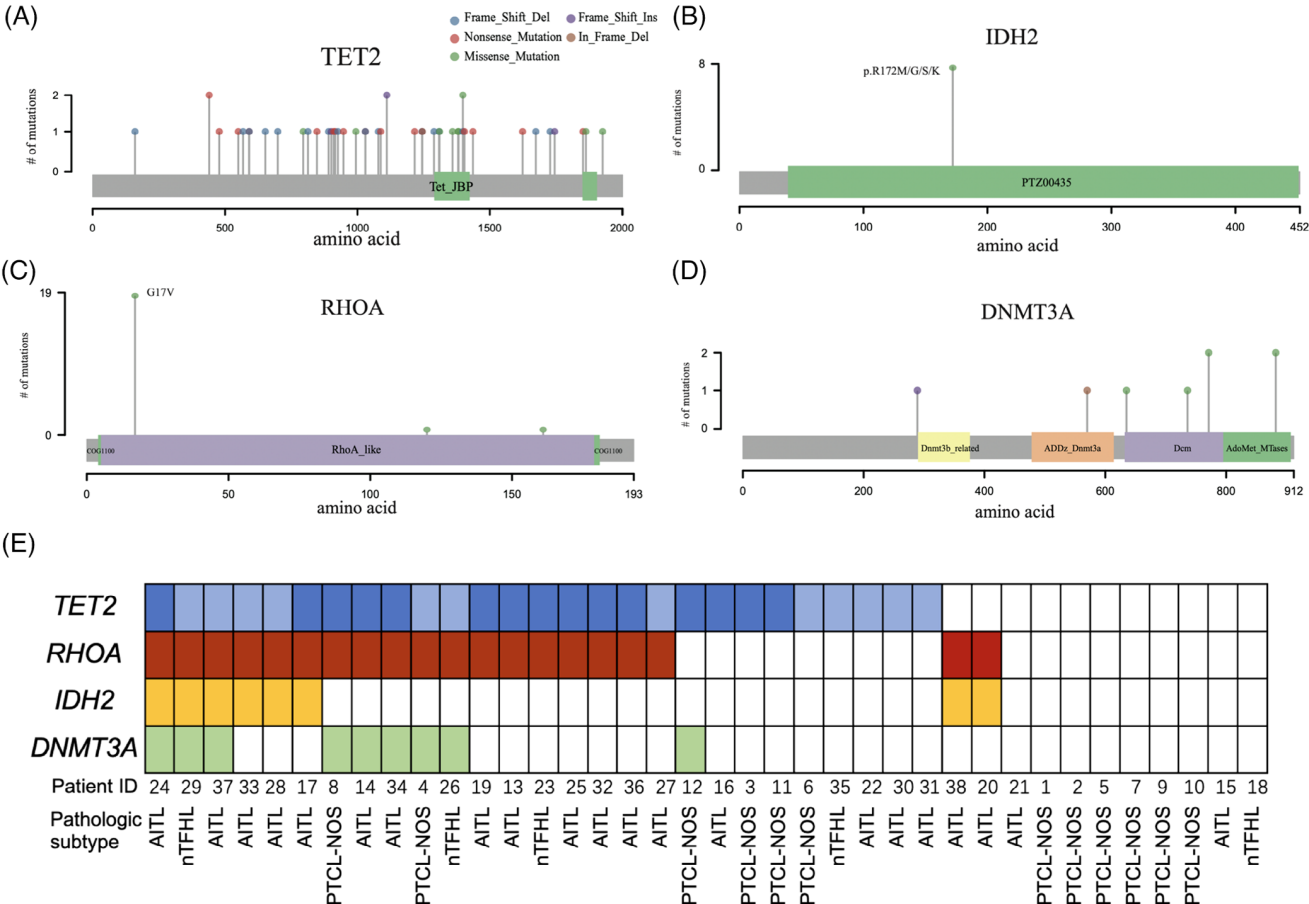
Mutation spectrum of the epigenetic regulators detected in the 38 patients with peripheral T‐cell lymphomas (PTCLs). (A–D) Lollipop plots representing the position of each mutation in *TET2*, *DNMT3A*, *IDH2* and *RHOA* genes. (E) Distribution of mutations in *TET2*, *RHOA*, *IDH2* and *DNMT3A* in the 38 PTCL samples. Light blue indicates samples having a single *TET2* mutation and dark blue indicates samples having multiple *TET2* mutations.

We next evaluated the impact of individual epigenetic mutations on treatment response. Responses were evaluable in 36 of the 38 patients. Among the epigenetic genes evaluated, only DNMT3A mutation was found to be associated with an adverse response rate. The mutation frequency of *DNMT3A* mutation was significantly higher in the non‐responder group than that in the responder group (mutation frequency of 41.2% vs. 5.3%, *p* = .016) (Figure 3A). Similarly, patients with mutated *DNMT3A* had significantly lower responses rate than those with wild‐type *DNMT3A* (ORR, 12.5% vs. 64.3%, *p* = .016; CR rate, .0% vs.57.1%, *p* = .005). In subgroup analysis stratified by treatment modalities (C‐CHOEP or AC‐CHOP), none of the epigenetic regulators showed significant association with the response with a small sample size in each subgroup (Figure 3B). Further, in subgroup analysis stratified by pathological subtypes (PTCL‐NOS or AITL/nTFHL), *DNMT3A* mutation maintained the prognostic value of poor response rate in the AITL/nTFHL subgroup (Figure 3C). The frequency of *DNMT3A* mutation was still significantly higher in the non‐responder than in the responder group (mutation frequency of 46.2% vs. 0%, *p* = .015) among patients with AITL and nTFHL. In previous studies, the correlation between *TET2* mutation status and the response to epigenetic treatment was the most studied and showed controversial results.^7^, ^10^ However, *TET2* mutation did not show a significant correlation with treatment response in the analysis of the entire group and subgroup analyses stratified by treatment modalities and pathological subtypes in our study.

**FIGURE 3:**
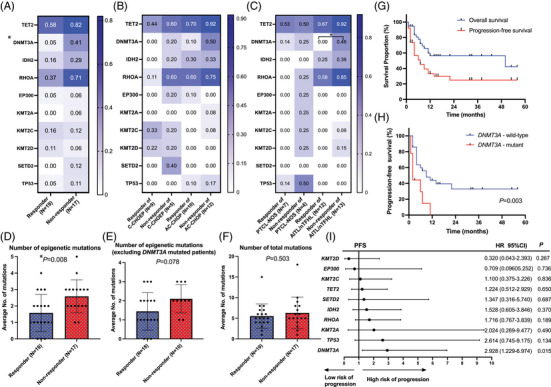
Correlation of epigenetic mutations with treatment response and survival. (A) Heatmap illustrating the mutation frequency of each epigenetic regulator between the responders and non‐responders. (B and C) Heatmap illustrating the mutation frequency of each epigenetic regulator between the responders and non‐responders stratified by treatment modalities (B) and pathological subtypes (C). (D) Comparison of the number of epigenetic mutations between the responders and non‐responders. (E) Comparison of the number of total somatic mutations between the responders and non‐responders. (F) Comparison of the number of epigenetic mutations between the responders and non‐responders excluding patients with *DNMT3A* mutations. (G) Progression‐free survival (PFS) and overall survival of the entire cohort. (H) Progression‐free survival of patients with mutant *DNMT3A* and wild‐type *DNMT3A*. (I) Forest plot of the hazard ratio of different epigenetic mutations in univariate analysis for PFS. Horizontal lines show the 95% CI for each factor.

Different types of epigenetic mutations may reshape the epigenome globally. To test this hypothesis, we further evaluated the correlation of the number of epigenetic mutations with treatment response. We found that non‐responding patients exhibited a higher average number of epigenetic mutations compared with the responders (2.6 vs. 1.6, *p* = .008), which suggests that a high mutation burden of epigenetic genes may predict a low response rate (Figure 3D). Additionally, the number of total somatic mutations between the responder and non‐responders was not significantly different, which indicated that response rates were truly associated with epigenetic mutational burden instead of global mutational burden (Figure 3E). When excluding patients with the *DNMT3A* mutations from analysis, a higher average number of epigenetic mutations was also found among the non‐responders than among the responders (2.1 vs. 1.4, *p* = .078) (Figure 3F). However, the difference did not approach statistical significance. This result was inconsistent with a previously published study by Falchi et al, in which responders harbored a higher average number of epigenetic mutations. However, the treatment modalities and treatment histories of enrolled patients were clearly different between this previous study and our study. In this sense, we still should be cautious against broad generalization of conclusions drawn from our study until they can be validated in larger‐scale controlled studies.

Finally, we assessed the correlation between epigenetic mutations and survival. The median follow‐up time was 16 (range: 1 ‐ 56) months. The median PFS and OS time were 7 and 50 months for the whole cohort (Figure 3G). The 1‐year and 2‐year PFS rates were 33.3% and 24.9%, respectively. The 1‐year and 2‐year OS rates were both 56.4%. In univariate analysis for PFS, established risk factors for PTCL including age, LDH level, ECOG status, Ann Arbor stage, extranodal sites involvement, bone marrow involvement along with epigenetic mutations of *TET2*, *IDH2*, *DNMT3A*, *KMT2A*, *KMT2C*, *KMT2D*, *SETD2* and *EP300* were incorporated. Only *DNMT3A* mutation was identified as an independent adverse prognostic factors for PFS in univariate analysis (*HR* = 2.928, 95% *CI*: 1.229‐6.974, *p* = .015) (Figure 3I). Patients with mutant *DNMT3A* showed significantly inferior 1‐ and 2‐year PFS rates compared with those with wild type (0% and 0% vs. 42.4% and 31.8%) (Figure 3H). In univariate analysis for OS, none of the above factors showed significant prognostic value. These results were consistent with a recently published study by Ruan J et al, in which DNMT3A mutation was also reported to be associated with adverse PFS for patients with PTCLs and treated with oral azacitidine plus CHOP.^7^ In another study evaluating histone modifier gene mutations in PTCL‐NOS, Ji et al reported that mutations of histone modifier genes were associated with inferior PFS for patients with PTCL‐NOS.^11^ However, in our study, both PFS and OS showed no significant differences between patients with or without histone modifier gene mutations (Figure S2)

This study, like many other studies on this relatively rare disease, the limitations included the small sample size, the heterogeneity of treatment modalities, and the usage of different NGS panels. Clonal hematopoiesis was not evaluated which may further influence the treatment efficacy and survival. Findings in our preliminary study can serve as a reference for large scale multicenter studies in the future.

In conclusion, widespread epigenetic mutations were identified in patients with PTCLs. *DNMT3A* mutation may serve as a potential biomarker in predicting resistance to chemotherapies priming with epigenetic targeting drugs and adverse PFS in patients with PTCLs. A high mutation burden of epigenetic genes may also predict poor treatment responses.

## AUTHOR CONTRIBUTIONS

CW, DBZ, and WZ designed the study. CW, WW, YZ, and DQZ performed the experiment. CW and WYL analyzed all the data. WZ and DBZ helped perform the analysis with constructive discussions. CW wrote the main manuscript. DBZ and WZ reviewed and revised the manuscript. All authors read and approved the final manuscript.

## FUNDING INFORMATION

National Natural Science Foundation of China (NSFC), Grant Number: 81970188; National High Level Hospital Clinical Research Funding, Grant Numbers: 2022‐PUMCH‐A‐261, 2022‐PUMCH‐C‐056, and 2022‐PUMCH‐B‐134

## CONFLICT OF INTEREST STATEMENT

The authors declare no potential conflict of interest.

## ETHICAL APPROVAL

This study was approved by the institutional review board of Peking Union Medical College Hospital and the study was conducted in accordance with the Declaration of Helsinki.

## CONSENT FOR PUBLICATION

Not applicable.

## Supporting information


**Figure S1**. Flowchart of patients’ enrollment. C‐CHOEP: chidamide combined with cyclophosphamide, doxorubicin, vincristine, prednisone and etoposide regimen; AC‐CHOP: chidamide, azacitidine combined with cyclophosphamide, doxorubicin, vincristine and prednisone regimen.

**Figure S2**. Progression‐free survival (A) and overall survival (B) of patients with or without histone modifier gene mutations.

Supporting Information

Supporting Information

## Data Availability

The data generated in this study are available upon request to the corresponding author.
