# Supplementary material for: Mutations of epigenetic genes and correlation with treatment response in peripheral T‐cell lymphoma
Source: Clin Transl Med. 2024 Jan 18;14(1):e1491. doi: 10.1002/ctm2.1491 (PMC10797249; doi:10.1002/ctm2.1491)
Supplement: Supplementary file 3 — Supporting Information [file CTM2-14-e1491-s002.docx]

**Table S1.** Gene information in the sequencing panel

| Gene symbol | Mutation type | | | |
| --- | --- | --- | --- | --- |
|  | CDS | SV | SNV | Hot region |
| ABCB1 | yes | - | - | - |
| ABL1 | yes | - | - | - |
| ABL2 | yes | - | - | - |
| ACTB | yes | - | - | - |
| AIM1 | yes | - | - | - |
| AKT1 | yes | - | - | - |
| AKT2 | yes | - | - | - |
| AKT3 | yes | - | - | - |
| ALK | yes | yes | - | - |
| ALOX12B | yes | - | - | - |
| ANKRD11 | yes | - | - | - |
| APC | yes | - | - | - |
| AR | yes | - | - | - |
| ARID1A | yes | - | - | - |
| ARID1B | yes | - | - | - |
| ARID2 | yes | - | - | - |
| ASXL1 | - | - | - | yes |
| ASXL3 | yes | - | - | - |
| ATG5 | yes | - | - | - |
| ATM | yes | - | - | - |
| ATP10B | yes | - | - | - |
| ATR | yes | - | - | - |
| ATRX | yes | - | - | - |
| AURKA | yes | - | - | - |
| AURKB | yes | - | - | - |
| AXIN1 | yes | - | - | - |
| AXL | yes | - | - | - |
| B2M | yes | - | - | - |
| BARD1 | yes | - | - | - |
| BCL10 | yes | - | - | - |
| BCL11B | yes | - | - | - |
| BCL2 | yes | yes | - | - |
| BCL6 | yes | yes | - | - |
| BCL7A | yes | - | - | - |
| BCOR | yes | - | - | - |
| BCORL1 | yes | - | - | - |
| BCR | - | yes | - | - |
| BIRC3 | yes | yes | - | - |
| BLM | yes | - | - | - |
| BRAF | yes | yes | - | - |
| BRCA1 | yes | - | - | - |
| BRCA2 | yes | - | - | - |
| BRD4 | yes | - | - | - |
| BRD9 | yes | - | - | - |
| BRIP1 | yes | - | - | - |
| BTG1 | yes | - | - | - |
| BTG2 | yes | - | - | - |
| BTK | yes | - | - | - |
| BUB1 | yes | - | - | - |
| BUB1B | yes | - | - | - |
| CACNA2D1 | yes | - | - | - |
| CALR | - | - | - | yes |
| CARD11 | yes | - | - | - |
| CBL | yes | - | - | - |
| CCND1 | yes | yes | - | - |
| CCND2 | yes | - | - | - |
| CCND3 | yes | - | - | - |
| CCR4 | yes | - | - | - |
| CCR7 | yes | - | - | - |
| CCT6B | yes | - | - | - |
| CD22 | yes | - | - | - |
| CD274(PDL1) | yes | yes | - | - |
| CD28 | yes | - | - | - |
| CD36 | yes | - | - | - |
| CD58 | yes | - | - | - |
| CD70 | yes | - | - | - |
| CD79A | yes | - | - | - |
| CD79B | yes | - | - | - |
| CDC73 | yes | - | - | - |
| CDK12 | yes | - | - | - |
| CDK4 | yes | - | - | - |
| CDK6 | yes | - | - | - |
| CDK8 | yes | - | - | - |
| CDKN1B | yes | - | - | - |
| CDKN2A | yes | - | - | - |
| CDKN2B | yes | - | - | - |
| CDKN2C | yes | - | - | - |
| CEBPA | yes | - | - | - |
| CHD2 | yes | - | - | - |
| CHD3 | yes | - | - | - |
| CHD8 | yes | - | - | - |
| CHEK1 | yes | - | - | - |
| CHEK2 | yes | - | - | - |
| CHPF2 | yes | - | - | - |
| CIC | yes | - | - | - |
| CIITA | yes | - | - | - |
| CKS1B | yes | - | - | - |
| CRBN | yes | - | - | - |
| CREBBP | yes | - | - | - |
| CRKL | yes | - | - | - |
| CRLF2 | - | - | - | yes |
| CSF1R | yes | - | - | - |
| CSF3R | yes | - | - | - |
| CSNK1A1 | yes | - | - | - |
| CSNK2A1 | yes | - | - | - |
| CSNK2B | yes | - | - | - |
| CTCF | yes | - | - | - |
| CTLA4 | - | yes | - | - |
| CTNNB1 | yes | - | - | - |
| CUL4A | yes | - | - | - |
| CUL4B | yes | - | - | - |
| CUX1 | yes | - | - | - |
| CXCR4 | yes | - | - | - |
| CXCR5 | yes | - | - | - |
| DAXX | yes | - | - | - |
| DDR2 | yes | - | - | - |
| DDX3X | yes | - | - | - |
| DDX41 | yes | - | - | - |
| DIS3 | yes | - | - | - |
| DNMT1 | yes | - | - | - |
| DNMT3A | yes | - | - | - |
| DNMT3B | yes | - | - | - |
| DTX1 | yes | - | - | - |
| DUSP2 | yes | - | - | - |
| DUSP22 | - | yes | - | - |
| EBF1 | yes | - | - | - |
| ECSIT | - | - | - | yes |
| ECT2L | yes | - | - | - |
| EGFR | yes | - | - | - |
| EIF4A1 | yes | - | - | - |
| EP300 | yes | - | - | - |
| EPHA3 | yes | - | - | - |
| EPHA5 | yes | - | - | - |
| EPHA7 | yes | - | - | - |
| ERBB2 | yes | - | - | - |
| ERBB3 | yes | - | - | - |
| ERBB4 | yes | - | - | - |
| ETS1 | yes | - | - | - |
| ETV6 | yes | yes | - | - |
| EZH2 | yes | - | - | - |
| FAF1 | yes | - | - | - |
| FAM46C | yes | - | - | - |
| FANCA | yes | - | - | - |
| FANCC | yes | - | - | - |
| FANCD2 | yes | - | - | - |
| FANCE | yes | - | - | - |
| FANCG | yes | - | - | - |
| FANCL | yes | - | - | - |
| FAS | yes | - | - | - |
| FAT3 | yes | - | - | - |
| FBXO11 | yes | - | - | - |
| FBXW7 | yes | - | - | - |
| FGF14 | yes | - | - | - |
| FGF19 | yes | - | - | - |
| FGF3 | yes | - | - | - |
| FGF4 | yes | - | - | - |
| FGFR1 | yes | - | - | - |
| FGFR2 | yes | - | - | - |
| FGFR3 | yes | - | - | - |
| FGFR4 | yes | - | - | - |
| FLT1 | yes | - | - | - |
| FLT3 | - | - | - | yes |
| FLT4 | yes | - | - | - |
| FOXC1 | yes | - | - | - |
| FOXO1 | yes | - | - | - |
| FOXO3 | yes | - | - | - |
| FOXP1 | yes | - | - | - |
| FRS2 | yes | - | - | - |
| FYN | yes | - | - | - |
| GADD45B | yes | - | - | - |
| GATA1 | yes | - | - | - |
| GATA2 | yes | - | - | - |
| GATA3 | yes | - | - | - |
| GNA11 | yes | - | - | - |
| GNA13 | yes | - | - | - |
| GNAQ | yes | - | - | - |
| GNAS | yes | - | - | - |
| GPR183 | yes | - | - | - |
| GRHPR | yes | - | - | - |
| GRIN2A | yes | - | - | - |
| HDAC1 | yes | - | - | - |
| HDAC4 | yes | - | - | - |
| HDAC7 | yes | - | - | - |
| HGF | yes | - | - | - |
| HIST1H1C | yes | - | - | - |
| HIST1H1D | yes | - | - | - |
| HIST1H1E | yes | - | - | - |
| HIST1H2AC | yes | - | - | - |
| HIST1H2AG | yes | - | - | - |
| HIST1H2AL | yes | - | - | - |
| HIST1H2AM | yes | - | - | - |
| HIST1H2BC | yes | - | - | - |
| HIST1H2BJ | yes | - | - | - |
| HIST1H2BK | yes | - | - | - |
| HIST1H3B | yes | - | - | - |
| HNF1A | yes | - | - | - |
| HNRNPA2B1 | yes | - | - | - |
| HRAS | yes | - | - | - |
| ID3 | yes | - | - | - |
| IDH1 | yes | - | - | - |
| IDH2 | yes | - | - | - |
| IGF1R | yes | - | - | - |
| IGH | - | yes | - | - |
| IKBKE | yes | - | - | - |
| IKZF1 | yes | - | - | - |
| IKZF2 | yes | - | - | - |
| IKZF3 | yes | - | - | - |
| IL7R | yes | - | - | - |
| INHBA | yes | - | - | - |
| INPP4B | yes | - | - | - |
| INPP5D | yes | - | - | - |
| INTS8 | yes | - | - | - |
| IRF1 | yes | - | - | - |
| IRF2BP2 | yes | - | - | - |
| IRF4 | yes | yes | - | - |
| IRF8 | yes | - | - | - |
| IRS2 | yes | - | - | - |
| ITK | yes | - | - | - |
| ITPKB | yes | - | - | - |
| JAK1 | yes | yes | - | - |
| JAK2 | yes | yes | - | - |
| JAK3 | yes | - | - | - |
| JUN | yes | - | - | - |
| KCNH8 | yes | - | - | - |
| KDM4C | yes | - | - | - |
| KDM5A | yes | - | - | - |
| KDM6A | yes | - | - | - |
| KDR | yes | - | - | - |
| KIT | yes | - | - | - |
| KLHL14 | yes | - | - | - |
| KLHL6 | yes | - | - | - |
| KMT2A(MLL) | yes | yes | - | - |
| KMT2B(MLL4) | yes | - | - | - |
| KMT2C(MLL3) | yes | - | - | - |
| KMT2D(MLL2) | yes | - | - | - |
| KRAS | yes | - | - | - |
| LMCD1 | yes | - | - | - |
| LRP1B | yes | - | - | - |
| LRRK2 | yes | - | - | - |
| MAF | yes | - | - | - |
| MAFB | yes | - | - | - |
| MALT1 | yes | - | - | - |
| MAP2K1 | yes | - | - | - |
| MAP2K2 | yes | - | - | - |
| MAP2K4 | yes | - | - | - |
| MAP3K1 | yes | - | - | - |
| MAP3K14 | yes | - | - | - |
| MAP3K6 | yes | - | - | - |
| MAP3K7 | yes | - | - | - |
| MAPK1 | yes | - | - | - |
| MCL1 | yes | - | - | - |
| MDM2 | yes | - | - | - |
| MDM4 | yes | - | - | - |
| MECOM | yes | - | - | - |
| MED12 | yes | - | - | - |
| MEF2B | yes | - | - | - |
| MEF2C | yes | - | - | - |
| MEN1 | yes | - | - | - |
| MET | yes | - | - | - |
| MGA | yes | - | - | - |
| MGAM | yes | - | - | - |
| MITF | yes | - | - | - |
| MLH1 | yes | - | - | - |
| MPEG1 | yes | - | - | - |
| MPL | yes | - | - | - |
| MSH2 | yes | - | - | - |
| MSH3 | yes | - | - | - |
| MSH6 | yes | - | - | - |
| MSN | yes | - | - | - |
| MTAP | yes | - | - | - |
| MTOR | yes | - | - | - |
| MUTYH | yes | - | - | - |
| MYB | yes | - | - | - |
| MYC | yes | yes | - | - |
| MYCL1 | yes | - | - | - |
| MYCN | yes | - | - | - |
| MYD88 | yes | - | - | - |
| MYH9 | yes | - | - | - |
| MYST3(KAT6A) | - | - | - | yes |
| NCOR2 | yes | - | - | - |
| NF1 | yes | - | - | - |
| NFKB2 | yes | - | - | - |
| NFKBIA | yes | - | - | - |
| NOTCH1 | yes | - | - | - |
| NOTCH2 | yes | - | - | - |
| NPM1 | yes | - | - | - |
| NRAS | yes | - | - | - |
| NT5C2 | - | - | - | yes |
| NTRK1 | - | yes | - | - |
| NTRK2 | yes | - | - | - |
| NTRK3 | yes | - | - | - |
| OSBPL10 | yes | - | - | - |
| P2RY8 | yes | - | - | - |
| PAK3 | yes | - | - | - |
| PALB2 | yes | - | - | - |
| PARP1 | yes | - | - | - |
| PAX5 | yes | - | - | - |
| PCBP1 | yes | - | - | - |
| PCLO | yes | - | - | - |
| PDCD1 | yes | - | - | - |
| PDCD11 | yes | - | - | - |
| PDCD1LG2(PDL2) | yes | yes | - | - |
| PDGFRA | yes | yes | - | - |
| PDGFRB | yes | yes | - | - |
| PDK1 | yes | - | - | - |
| PHF6 | yes | - | - | - |
| PIK3CA | yes | - | - | - |
| PIK3CD | yes | - | - | - |
| PIK3CG | yes | - | - | - |
| PIK3R1 | yes | - | - | - |
| PIK3R2 | yes | - | - | - |
| PIM1 | yes | - | - | - |
| PIM2 | yes | - | - | - |
| PLCG1 | yes | - | - | - |
| PLCG2 | yes | - | - | - |
| PMS2 | yes | - | - | - |
| POT1 | yes | - | - | - |
| PPP2R1A | yes | - | - | - |
| PRDM1 | yes | - | - | - |
| PRKCB | yes | - | - | - |
| PRKCQ | yes | - | - | - |
| PRKDC | yes | - | - | - |
| PRKG1 | yes | - | - | - |
| PTCH1 | yes | - | - | - |
| PTEN | yes | - | - | - |
| PTPN11 | yes | - | - | - |
| PTPN2 | yes | - | - | - |
| PTPN6 | yes | - | - | - |
| PTPRD | yes | - | - | - |
| RAD50 | yes | - | - | - |
| RAD51 | yes | - | - | - |
| RAF1 | yes | - | - | - |
| RARA | yes | yes | - | - |
| RB1 | yes | - | - | - |
| REL | yes | - | - | - |
| RELN | yes | - | - | - |
| RET | yes | - | - | - |
| RFXAP | yes | - | - | - |
| RGS7 | yes | - | - | - |
| RHOA | yes | - | - | - |
| RICTOR | yes | - | - | - |
| ROS1 | yes | yes | - | - |
| RPTOR | yes | - | - | - |
| RUNX1 | yes | - | - | - |
| RUNX1T1 | yes | - | - | - |
| S1PR2 | yes | - | - | - |
| SETBP1 | yes | - | - | - |
| SETD1B | yes | - | - | - |
| SETD2 | yes | - | - | - |
| SF3B1 | yes | - | - | - |
| SGK1 | yes | - | - | - |
| SH2B3 | yes | - | - | - |
| SH2D1A | yes | - | - | - |
| SMAD2 | yes | - | - | - |
| SMAD4 | yes | - | - | - |
| SMARCA4 | yes | - | - | - |
| SMARCB1 | yes | - | - | - |
| SMC1A | yes | - | - | - |
| SMC3 | yes | - | - | - |
| SMO | yes | - | - | - |
| SOCS1 | yes | - | - | - |
| SOCS2 | yes | - | - | - |
| SOX10 | yes | - | - | - |
| SOX11 | yes | - | - | - |
| SOX2 | yes | - | - | - |
| SPEN | yes | - | - | - |
| SPIB | yes | - | - | - |
| SPOP | yes | - | - | - |
| SRC | yes | - | - | - |
| SRSF2 | yes | - | - | - |
| STAT3 | yes | - | - | - |
| STAT5A | yes | - | - | - |
| STAT5B | yes | - | - | - |
| STAT6 | yes | - | - | - |
| STK10 | yes | - | - | - |
| STK11 | yes | - | - | - |
| STX11 | yes | - | - | - |
| STXBP2 | yes | - | - | - |
| SUZ12 | yes | - | - | - |
| SYK | yes | yes | - | - |
| TAF1 | yes | - | - | - |
| TBL1XR1 | yes | - | - | - |
| TCF3 | yes | yes | - | - |
| TCL1A | yes | - | - | - |
| TERT | - | - | - | yes |
| TET1 | yes | - | - | - |
| TET2 | yes | - | - | - |
| TGFBR2 | yes | - | - | - |
| TLX3 | yes | - | - | - |
| TMEM30A | yes | - | - | - |
| TNFAIP3 | yes | - | - | - |
| TNFR2(TNFRSF1B) | yes | - | - | - |
| TNFRSF11A | yes | - | - | - |
| TNFRSF14 | yes | - | - | - |
| TNFRSF17 | yes | - | - | - |
| TOX | yes | - | - | - |
| TP53 | yes | - | - | - |
| TP63 | yes | yes | - | - |
| TP73 | yes | - | - | - |
| TPMT | - | - | yes | - |
| TRAF2 | yes | - | - | - |
| TRAF3 | yes | - | - | - |
| TRAF5 | yes | - | - | - |
| TRMT12 | yes | - | - | - |
| TRRAP | yes | - | - | - |
| TSC1 | yes | - | - | - |
| TSC2 | yes | - | - | - |
| TSHR | yes | - | - | - |
| U2AF1 | yes | - | - | - |
| UBE2A | yes | - | - | - |
| VAV1 | yes | - | - | - |
| WDR17 | yes | - | - | - |
| WHSC1 | yes | - | - | - |
| WT1 | yes | - | - | - |
| WWOX | yes | - | - | - |
| XBP1 | yes | - | - | - |
| XIAP | yes | - | - | - |
| XPO1 | yes | - | - | - |
| ZAP70 | yes | - | - | - |
| ZEB1 | yes | - | - | - |
| ZNF217 | yes | - | - | - |
| ZP4 | yes | - | - | - |

Abbreviations: CDS: coding sequence; SV: structure variation; SNP: single nucleotide polymorphis.
