# Supplementary material for: Mutations of epigenetic genes and correlation with treatment response in peripheral T‐cell lymphoma
Source: Clin Transl Med. 2024 Jan 18;14(1):e1491. doi: 10.1002/ctm2.1491 (PMC10797249; doi:10.1002/ctm2.1491)
Supplement: Supplementary file 4 — Supporting Information [file CTM2-14-e1491-s004.docx]

**Table S2.** Gene information in the sequencing panel

| Gene symbol | Mutation type | | | |
| --- | --- | --- | --- | --- |
|  | CDS | SV | SNV | Hot region |
| ALK | yes | - | - | - |
| APC | yes | - | - | - |
| APC2 | yes | - | - | - |
| ARID1A | yes | - | - | - |
| ARID1B | yes | - | - | - |
| ARID2 | yes | - | - | - |
| ASXL3 | yes | - | - | - |
| ATM | yes | - | - | - |
| BCOR | yes | - | - | - |
| BIRC3 | yes | - | - | - |
| BIRC6 | yes | - | - | - |
| CARD11 | yes | - | - | - |
| CCND3 | yes | - | - | - |
| CD58 | yes | - | - | - |
| CDKN2A | yes | - | - | - |
| CHD8 | yes | - | - | - |
| CHEK2 | yes | - | - | - |
| CIC | yes | - | - | - |
| CIITA | yes | - | - | - |
| CREBBP | yes | - | - | - |
| DNMT3A | yes | - | - | - |
| EP300 | yes | - | - | - |
| FYN | yes | - | - | - |
| HLA-A | yes | - | - | - |
| HLA-B | yes | - | - | - |
| IDH2 | yes | - | - | - |
| IKBKB | yes | - | - | - |
| ITPKB | yes | - | - | - |
| ITPR3 | yes | - | - | - |
| JAK1 | yes | - | - | - |
| JAK2 | yes | - | - | - |
| JAK3 | yes | - | - | - |
| JMY | yes | - | - | - |
| KDM6B | yes | - | - | - |
| KMT2A | yes | - | - | - |
| KMT2C | yes | - | - | - |
| KMT2D | yes | - | - | - |
| LRP1B | yes | - | - | - |
| MEF2A | yes | - | - | - |
| MGA | yes | - | - | - |
| MSH2 | yes | - | - | - |
| MSH3 | yes | - | - | - |
| MSH6 | yes | - | - | - |
| MTOR | yes | - | - | - |
| NCOR2 | yes | - | - | - |
| NF1 | yes | - | - | - |
| NOTCH1 | yes | - | - | - |
| NOTCH2 | yes | - | - | - |
| NOTCH3 | yes | - | - | - |
| PDCD1 | yes | - | - | - |
| PHLPP1 | yes | - | - | - |
| PIK3R1 | yes | - | - | - |
| PIK3R2 | yes | - | - | - |
| PLCG1 | yes | - | - | - |
| PLCG2 | yes | - | - | - |
| PMS1 | yes | - | - | - |
| PTEN | yes | - | - | - |
| PTPN13 | yes | - | - | - |
| PTPRC | yes | - | - | - |
| PTPRD | yes | - | - | - |
| PTPRS | yes | - | - | - |
| REV3L | yes | - | - | - |
| RHOA | yes | - | - | - |
| SALL3 | yes | - | - | - |
| SETBP1 | yes | - | - | - |
| SETD1B | yes | - | - | - |
| SETD2 | yes | - | - | - |
| SMARCA2 | yes | - | - | - |
| SMARCA4 | yes | - | - | - |
| SOCS1 | yes | - | - | - |
| SPEN | yes | - | - | - |
| STAT3 | yes | - | - | - |
| STAT5B | yes | - | - | - |
| TAL1 | yes | - | - | - |
| TET1 | yes | - | - | - |
| TET2 | yes | - | - | - |
| TET3 | yes | - | - | - |
| TP53 | yes | - | - | - |
| TRRAP | yes | - | - | - |
| TSC2 | yes | - | - | - |
| VAV1 | yes | - | - | - |
| YEATS2 | yes | - | - | - |
| YTHDF2 | yes | - | - | - |
| ZEB1 | yes | - | - | - |

Abbreviations: CDS: coding sequence; SV: structure variation; SNP: single nucleotide polymorphis.
